# Removal of tetracycline from wastewater using pumice stone: equilibrium, kinetic and thermodynamic studies

**DOI:** 10.1186/2052-336X-12-79

**Published:** 2014-05-01

**Authors:** Ulker Asli Guler, Meltem Sarioglu

**Affiliations:** 1 Department of Environmental Engineering, Engineering Faculty, Cumhuriyet University, Sivas 58140, Turkey

**Keywords:** Tetracyline, Antibiotic, Adsorption, Pumice stone, Na^+^, Cu^2+^

## Abstract

In this study, pumice stone was used for the removal of tetracyline (TC) from aqueous solutions. It was characterized by XRD, FT-IR, SEM and BET analyses. Cation exchange capacity of pumice stone was found to be 9.9 meq/100 g. Effect of various parameters such as solution pH (2–11), adsorbent dosage (0.5-10 g/L), contact time (2.5-120 min), initial TC concentration (5–300 mg/L) and temperature (20–50°C) on TC adsorption onto pumice was investigated. Also the adsorption of TC on pumice stone was studied as a function of Na^+^ and Cu^2+^ cations changing pH from 2 to 11 using batch experiments. The best removal efficiency performance was exhibited at adsorbent dosage 10 g/L, pH 3, contact time 120 min. Langmuir, Freundlich and Dubinin-Radushkevich (D-R) isotherm models were applied to the equilibrium data. The result has shown that the adsorption was favorable, physicochemical in nature and agrees well with Langmuir and Freundlich models. The maximum Langmuir adsorption capacity was found to be 20.02 mg/g. The adsorption behavior of TC on pumices stone was fitted well in the pseudo-second order kinetics model. Thermodynamic parameters calculated from the adsorption data at different temperature showed that the adsorption reaction was feasible, spontaneous and exothermic.

## Introduction

Antibiotics are used the worldwide in human and veterinary medicine for about 70 years [1-3]. TCs are the second most common antibiotic family in both production and usage in the world [4]. TCs are relatively poorly absorbed by humans and animals. Large fractions of antibiotics including TC are excreted through urine and feces as unmodified main compound [3,5-9].

Residues of TC are frequently detected in soil and various environmental water samples such as surface water, ground water and drinking water [1,3,5,10-12]. The presence of residual antibiotics in soil and water is potentially hazardous for the bacteria and non-target organisms and these can promote the selection of genetic variants of microorganisms resulting in the occurrence of antibiotic resistant pathogens [13-17].

**Figure 1:**
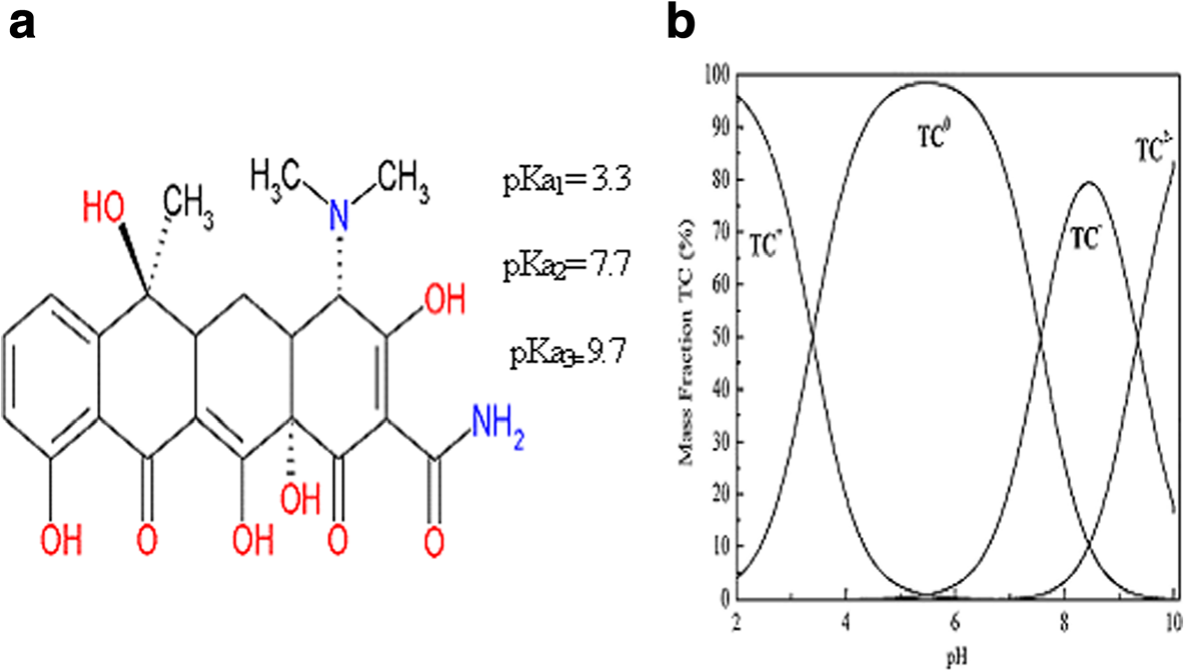
Chemical structure (a) and the speciation diagram (b) of TC as a function of pH.

The chemical structure of TC and the speciation diagram of TC as a function of pH are given in Figure 1a and Figure 1b, respectively. The pKa values of TC are 3.3, 7.7 and 9.7 [18,19].

TC molecule is strongly polar and has three protonactive groups: a dimethylamine (C-4), a tricarbonylamide group (C-1:C-2:C-3) and a phenolic diketone groups (C-10:C-11:C-12) (Figure 1) [3,9,20,21].

Many adsorbent has been used for TC removal from wastewaters; in this study the pumice stone was used for TC removal from wastewaters with comprehensive research.

TCs are highly adsorbed by several materials such as clay, montmorillonite, rectorite, palygorskite, chitosan particles, oxide minerals, humic substances, soil, activated carbon and sediments [3,7,15,17,20,22-29].

Pumice is a light, porous and volcanic stone with a large surface area. It is generally pale in color, ranging from white, cream, blue or grey, to green-brown or black [30] and used as an adsorbent, filter bed and support material in water and wastewater treatment [31-33].

In this study, effects of solution pH, adsorbent dosage, temperature, Na^+^ and Cu^2+^ cations on the TC adsorption by pumice stone were investigated. The adsorption isotherms, kinetics and thermodynamics were studied by batch experiments. In addition, the experimental results were combined with XRD, FT-IR and SEM analyses in order to identify in the interaction between pumice stone and TC and the specific surface area and components of pumice stone were determined by BET and XRF analyses, respectively.

## Materials and methods

### Adsorbent

Pumice stone was supplied from Kayseri-Basakpınar in Turkey. It was washed with distilled water several times and dried at 50°C in oven. Later, particle size of dried pumice stone was grinded to 0.125 mm below. The physicochemical properties and XRF analysis of pumice stone are listed in Table 1 and Table 2, respectively. Cation Exchange Capacity (CEC) was determined through ion exchange of sodium and potassium ions in the effluent.

**Table 1:** Properties of pumice stone

**Adsorbent**	**Details**	**Specific surface area (m**^**2**^**g**^**−1**^**)**	**Total pore volume (cm**^**3**^**g**^**−1**^**)**	**Mean pore diameter (nm)**	**pH**_**pzc**_	**CEC (meq/100 g)**
Pumice stone	Light grey/brown granular	11.88	0.0410	13.813	8.34	9.9

**Table 2:** XRF analysis of pumice stone

**Compound**	**% by weight**	**Compound**	**% by weight**
SiO_2_	69.27	P_2_O_5_	0.08
Al_2_O_3_	14.24	MnO	0.07
K_2_O	3.89	BaO	0.06
Na_2_O	3.61	ZrO_2_	0.05
Fe_2_O_3_	2.90	Cr_2_O_3_	0.01
CaO	1.82	ZnO	0.01
MgO	0.49	Loss on ignition	2.95
TiO_2_	0.45	**Total**	**100.00**
SO_3_	0.10		

### Batch experiments

The batch experiments were carried out in 250 mL Erlenmeyer containing 100 mL of aqueous solution. pH was adjusted with HCl and NaOH. The suspension was shaken in temperature controlled shaker at 130 rpm. The residual concentration of TC in supernatant was analysed by CHEBIOUS UV-spectrophotometer at λ_max_ value of TC (357 nm wavelength). Effects of solution pH (2.0-11), adsorbent dosage (0.5-10 g/L), contact time (2.5-120 min), initial TC concentration (5–300 mg/L) and temperature (20–50°C) on adsorption of TC by pumice stone were investigated. In addition, the effects of Na^+^ and Cu^2+^ cations on the TC adsorption were investigated as a function of pH. The details of the experimental conditions are presented in Table 3.

**Table 3:** Experimental conditions

**Experimental conditions**						
**Set**	**Aim of experiment**	**Solution pH**	**TC conc. (mg/L)**	**Adsorbent dosage (g/L)**	**Contact time (min)**	**Temperature**
1	Effect of solution pH	2.0-11.0	50	10	120	Room temperature (20°C)
2	Effect of adsorbent dosage	3.0	50	0.5-10	120	Room temperature (20°C)
3	Adsorption kinetics	3.0	50	10	2.5-120	Room temperature (20°C)
4	Adsorption equilibrium tests	3.0	5-300	10	120	Room temperature (20°C)
5	Effect of temperature	3.0	50	10	120	20-50°C
6	Effects of 0.01 M Na^+^ and 0.1 mM Cu^2+^	2.0-11.0	50	10	120	Room temperature (20°C)

The adsorption capacity (q_e_, mg/g) and removal efficiency (%) were determined with following equations:

(1) $$q_{e}=\frac{\left(C_{o}-C_{e}\right)V}{m}$$

(2) Removalefficiency%=Co−CeCo×100

where *C*_*o*_ and *C*_e_ are the initial and the equilibrium TC concentration (mg/L), *V* is the volume of solution (L) and *m* is the amount of pumice stone (g).

## Results and discussion

### Adsorbent characterization

**Figure 2:**
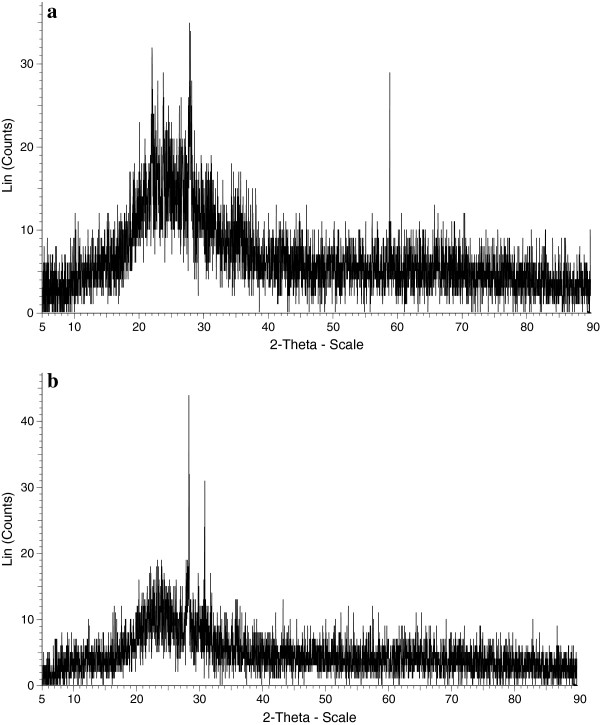
XRD patterns of pumice stone (a) and TC-P (b).

**Figure 3:**
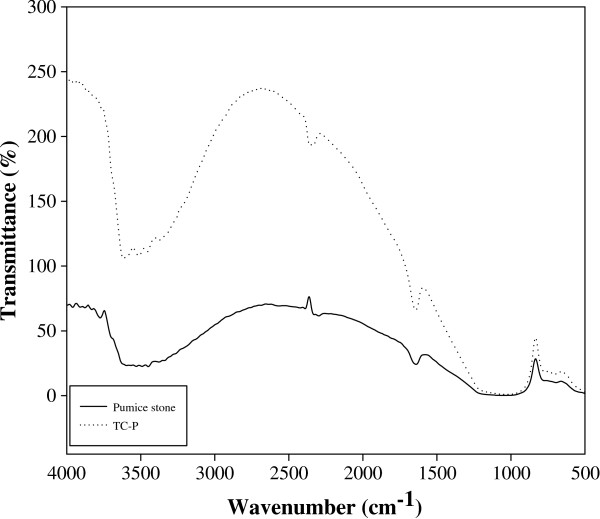
FT-IR spectra of pumice stone and TC-P.

**Figure 4:**
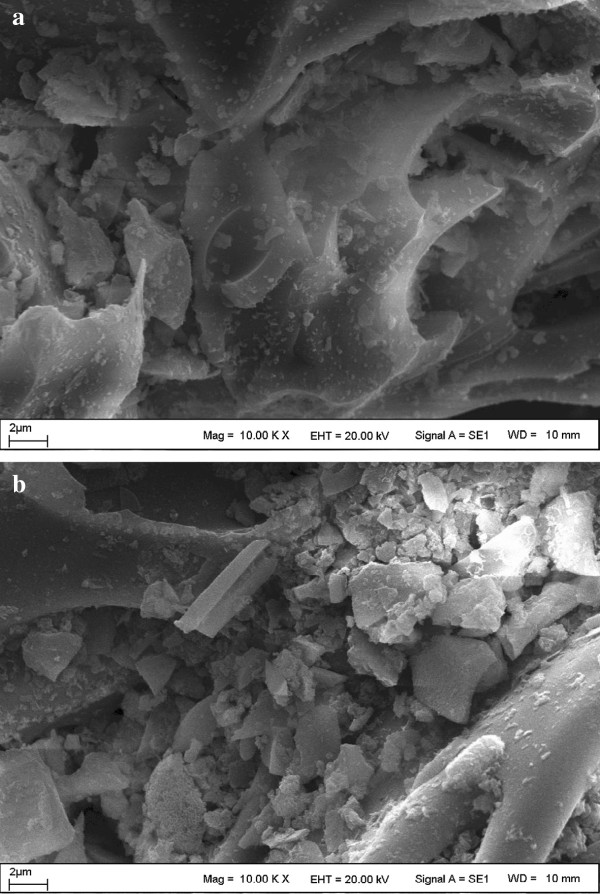
SEM images of pumice stone (a) and TC-P (b).

XRD, FT-IR and SEM analyses of pumice stone before and after (TC-P) the adsorption of TC are shown in Figures 2, 3 and 4, respectively.

According to the result of XRD pattern of the pumice stone, there were three peaks at 22, 28 and 59° (Figure 2). These peaks related to the mineral dachiardite (Ca, Na, K, Al, Si, H_2_O). The 100 peak of the quartz is known to be at 2θ = 26.65°. This finding points out amorphous quartz substance [34]. Similar results were also observed with the studies of other researchers [34-36]. The peaks of TC-P were observed at 28 and 31°. The changes of peaks are evidence of TC adsorption. As can be seen from XRF results, the two most important components of the pumice stone were SiO_2_ (69.27%) and Al_2_O_3_ (14.24%) (see Table 1). It can be concluded that the higher the silica percentage, the purer the pumice stone will be [34].

The most characteristic peaks of pumice stone and TC-P are 800, 1700 and 3500 cm^−1^. The peak at 800 cm^−1^ in FT-IR spectra of the pumice stone (Figure 3) may have resulted from the Si-O bending strength vibrations of the amorphous quartz. The peak at 1700 cm^−1^ can be assigned to amide I or C = O amide stretching and the amide group played an important role in TC adsorption. The other peak was observed around 3500 cm^−1^. This peak indicates the OH stretching vibrations of the adsorbed water (moisture) by the pumice stone from the outside environment [34,37]. These peaks were also determined with prior studies [34,37,38].

As can seen be from FT-IR spectra of TC-P, the peak at 1700 cm^−1^ of TC-P were observed different from the natural pumice stone. The band changes of amide I or C = O amide groups at 1700 cm^−1^ demonstrated that TC was adsorbed to the pumice stone with cation exchange and surface complexation [39].

The SEM images of natural pumice stone and TC-P indicated that the surface of pumice stone had a large porous surface and external surface of pumice stone was covered by TC, respectively (Figure 4). In addition, the specific surface area, total pore volume and mean pore diameter of pumice stone were 11.88 m^2^g^−1^, 0.0410 cm^3^g^−1^ and 13.813 nm, respectively.

### Effects of solution pH and Na^+^ and Cu^2+^ cations on adsorption

**Figure 5:**
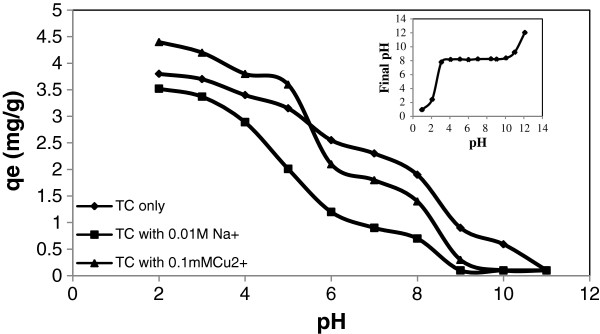
**Effect of pH, Na**^**+**^**, Cu**^**2+ **^**on TC adsorption and pH**_**pzc.**_

The effects of solution pH and Na^+^ and metal cation (Cu^2+^) on the TC adsorption onto pumice stone were investigated and results are shown in Figure 5. It can be seen that the adsorption of TC onto pumice stone was highly affected with the solution pH. When the solution pH was less than pKa_2_ (7.7) of TC, adsorption capacity of pumice stone varied from 3.80 mg/g to 2.30 mg/g at initial concentrations of 50 mg/L (Figure 5). Above pKa_2_, TC adsorption capacity of pumice stone decreased sharply to 0.59 mg/g at pH 10. This pH 10 and above there was almost no removal of TC from solution any more. Similar results were reported for TC adsorption onto graphene oxide and humic acid by other researches [3,9].

The zero point of charge (pH_pzc_) of pumice stone was found to be 8.34 (Figure 5). According to PZC value, pumice stone was positively charged at pH < 8.34 and negatively charged at pH > 8.34. The pKa values of TC molecule were 3.3, 7.7 and 9.27, respectively [1,40]. TC existed as cationic in strong acid solution at pH < 3.3, zwitter anions at 3.3 < pH < 7.7 and negative ions at pH > 7.7 [1,40,41]. In this case, adsorption mechanisms depending on pH were given in Table 4.

**Table 4:** Adsorption mechanisms depending on pH values

**Studied pH range**	**Adsorption Mechanisms**
2.0 < pH < 3.0	Cation exchange
3.0 < pH < 7.0	Cation exchange and surface complexation
pH 8.0	Surface complexation

Positively charge pumice stone < pH_pzc_ < negatively charge pumice stone.

The cation exchange mechanism between cations of TC and positively charged pumice stone surface was dominated at low pH values. A surface complexation mechanism for zwitter anion of TC was important, which was accompanied with proton uptake on pumice stone [19,23,42]. Physical mechanisms such as van der Waals forces attraction and hydrogen bonding between polar TC groups and acidic groups on the surface of pumice stone may also contribute to surface complexation mechanism in TC adsorption [22,23]. The decrease in TC removal under alkaline conditions may be due to competition of excess hydroxyl ions with anion TC for active sites on pumice stone [30]. The pH_PZC_ values of pumice stone in literature ranged between 6.9 and 9.3 [32]. The PZC value found in study is compatible with literature.

In order to determine the effects of Na^+^ and Cu^2+^ ions on the TC adsorption, 0.1 mM Cu^2+^ and 0.01 M Na^+^ cations were simultaneously adsorbed with TC at initial concentration of 50 mg/L. The results of TC adsorption by pumice stone in the presence of Na^+^ and Cu^2+^ was nearly similar. However, the presence of Cu^2+^ slightly increased TC adsorption on pumice stone at low pH (pH < 5). As a transitional metal cation, Cu^2+^ could form strong complexes both with TC and pumice stone [1,39]. Therefore, Cu^2+^ may role as a bridging ion on TC adsorption. The presence of Cu^2+^ had only minor effects on TC adsorption on pumice stone at high pH due to the formation of CuOH and Cu(OH)_2_[1]. Similar observations were reported on the TC adsorption onto soil and sediment in the presence of copper [1]. On the other hand, the presence of Na cation decreased on TC adsorption. This condition can be explained as the results of an electrostatic competition between TC cations and Na^+^ cations for adsorption to the same binding sites on surface of pumice stone. Similar results were also observed by other researchers [3,4].

In addition, the results were expressed with adsorption partition coefficient (K_D_ (L/eq)) defined in below [18,19]:

(3) $$K_{D}=\frac{q_{e}}{C_{e}\times \mathrm{CEC}}$$

**Figure 6:**
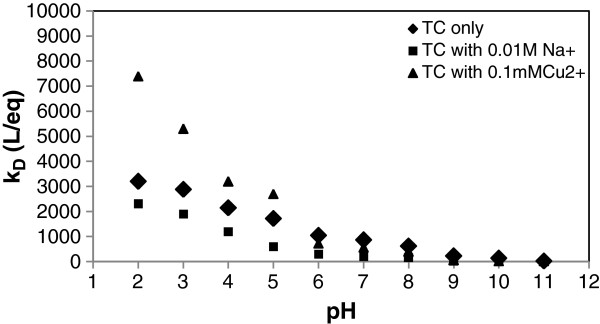
**The plot of K**_**D **_**against pH.**

The plot of K_D_ against pH is presented in Figure 6.

The adsorption partition coefficient is expressed with respect to the CEC because interactions of the TC with specific charge sites on surface of the pumice stone were anticipated [18]. The adsorption partition coefficients (K_D_) were relatively high at pH 2 and 3 and decreased at pH 4–9 and remained nearly constant between pH 9–11. The results of adsorption capacity (qe) and adsorption partition coefficients (K_D_) are compatible with each other and K_D_ and q_e_ values decreased with increasing pH value. In this case, the pH value was selected as 3 in further studies.

### Effect of adsorbent dosage on adsorption

**Figure 7:**
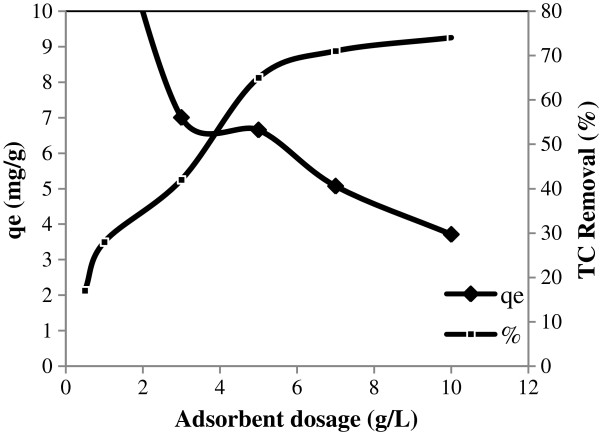
Effect of adsorbent dosage on TC adsorption.

The effect of adsorbent dosage on TC adsorption was studied in the range of 0.5-10 g/L. The TC removal and adsorption capacity at various adsorbent dosages are presented in Figure 7. According to Figure 7, TC percentage removal from 17% to 74% increased with increasing the adsorbent dosage from 0.5 to 10 g/L. The increase of TC adsorption is due to the availability of active binding sites and to the presence of a greater surface area for adsorption [30]. Therefore, the optimum dosage of pumice stone for further experiments of TC adsorption was selected as 10 g/L.

### Kinetics of TC adsorption onto pumice stone

**Figure 8:**
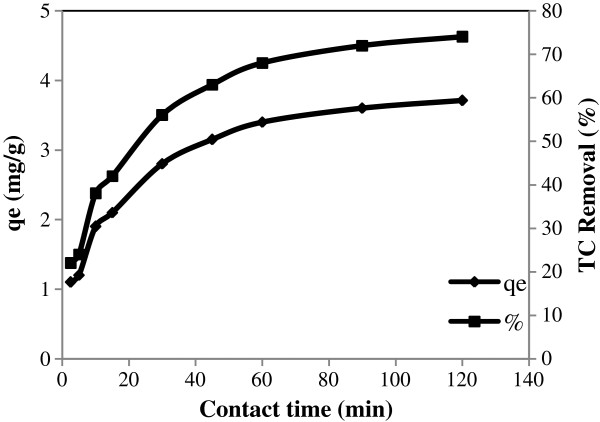
Effect of contact time on TC adsorption.

The effect of contact time in the range of 2.5-120 min and kinetics were calculated. The results regarding TC removal and adsorption capacity are presented in Figure 8.

**Figure 9:**
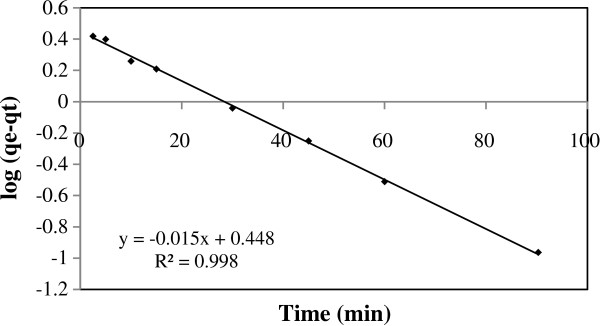
The pseudo first-order kinetic model of TC adsorption on pumice stone.

**Figure 10:**
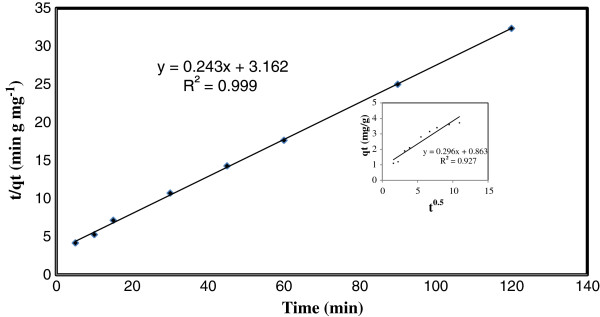
The pseudo second-order kinetic model and the intraparticle diffusion model of TC adsorption on pumice stone.

As shown in this figure, the fifty percent of TC adsorption occurred in the first 30 min and then the adsorption gradually reached equilibrium after 90 min contact time. The kinetic of TC adsorption on pumice stone was analyzed using the pseudo first-order Lagergren, pseudo second-order model and intraparticle diffusion model (Figures 9 and 10).

The pseudo first-order kinetic model of Lagergren is given as [30]:

(4) $$\log\left(q_{e}-q_{t}\right)=\log q_{e}-\frac{k_{1}}{2.303}t$$

The pseudo second-order kinetic model is expressed as [30]:

(5) $$\frac{t}{q_{t}}=\frac{1}{k_{2}{\left(q_{e}\right)}^{2}}+\frac{t}{q_{e}}$$

The intraparticle diffusion model equation can be described as [43]:

(6) $$q_{t}=k_{d}t^{0.5}+C$$

where q_e_ (mg/g) and q_t_ (mg/g) are the amounts of adsorbed TC on pumice stone at equilibrium and time t (min), C is the intercept and *k*_*1*_ (min^-1^), *k*_*2*_ (g/mg.min) and k_d_ (mg/g.min^0.5^) are the rate constant of pseudo first-order kinetic model, pseudo second-order kinetic model and intraparticle diffusion model, respectively. A straight line of log *(q*_*e*_*-q*_*t*_*)* versus *t* suggest the applicability of pseudo first-order kinetic model. *q*_*e*_ and *k*_*1*_ can be determined from the intercept and slope of the plot, respectively. The plot *t/q*_*t*_ versus *t* should give a straight line if pseudo second-order kinetics are applicable and *q*_*e*_ and *k*_*2*_ can be determined from the slope and intercept of the plot, respectively. The intraparticle diffusion model rate constat (k_d_) and C can be evaluated from the slope and intercept of the linear plot of q_t_ versus t^0.5^, respectively [44,45]. According to this intraparticle diffusion model, the plot of q_t_ versus t^0.5^ should be linear (C = 0) if intraparticle diffusion is involved in the overall adsorption mechanism and the intraparticle diffusion is the sole rate controlling step of the process. If this line did not through the origin (C ≠ 0), there are intraparticle diffusion and boundary layer effect in adsorption process. As the intercept value (C) increase, the effect of surface sorption in the rate controlling step increase [46].

The following expression denotes the initial sorption rate h (mg/g min):

(7) $$h=k_{2}{q_{e}}^{2}$$

The values of correlation coefficients (k_1_,k_2_, k_d_), equilibrium adsorption capacities (q_e,teo_) in these models (pseudo first-order kinetic model, pseudo second-order kinetic model and intraparticle diffusion model) and initial sorption rate (h) are given in Table 5.

**Table 5:** Kinetic parameters of the TC adsorption

	**Pseudo-first order**			**Pseudo-second order**			**Intra particle**			**h (mg/g.min)**
q_e,exp_	R^2^	k_1_ (min^-1^)	q_e, cal_	R^2^	k_2_ (g/mg.min)	q_e, cal_	R^2^	k_d_ (mg/g.min^0.5^)	C	0.34
3.71	0.998	0.03	2.80	0.999	0.02	4.11	0.927	0.30	0.87	

According to the fitted linear regression plots, the experimental data are well fitted to the pseudo-second order kinetic model with higher value correlation coefficient (R^2^ > 0.999) compared to pseudo-first order kinetic model. Kinetics of TC adsorption on pumice stone followed the pseudo-second order model, suggesting that the adsorption rate limiting step may be chemisorptions and the adsorption of TC occurs probably via surface complexation reactions at specific adsorption sites [20,30,47]. In addition, present study, the plots indicated that the intraparticle diffusion model was not the sole rate controlling step due to did not pass through the origin (C ≠ 0). This indicated that both intra particle diffusion and boundary diffusion affected the TC adsorption on pumice stone.

### Adsorption isotherms

In this study, the Langmuir, Freundlich and D-R adsorption models were used to describe the adsorption equilibrium. All isotherm models parameters were calculated by non-linear regression by using Sigmaplot 11 software. The equation of the Langmuir model is given [48]:

(8) $$q_{e}=\frac{Q_{m}bC_{e}}{1+bC_{e}}$$

where, *Q*_*m*_ (mg/g) is the maximum adsorption capacity and *b* (L/mg) is the Langmuir constant related to the affinity between adsorbent and sorbate. The essential feature of the Langmuir isotherm can be expressed in terms of R_L_, a dimensionless constant referred to as separation factor or equilibrium parameter. R_L_ is calculated using the following equation [49]:

(9) $$R_{L}=\frac{1}{1+bC_{o}}$$

The value of R_L_ indicates the type of the isotherm to be irreversible (R_L_ = 0), favorable (0 < R_L_ > 1), linear (R_L_ = 1) or unfavorable (R_L_ > 1).

The Freundlich isotherm is derived to model multilayer adsorption and adsorption on heterogeneous surfaces. The Freundlich isotherm is given by equation [48]:

(10) $$q_{e}=k_{F}{C_{e}}^{\frac{1}{n}}$$

where, *k*_*F*_ (L/g) is the Freundlich adsorption constant related to the adsorption capacity. *n* is the adsorption intensity. The *1/n* values were between 0 and 1 indicating that the adsorption was favorable at studied conditions.

D-R isotherm is more general than the Langmuir isotherm. It was applied to separate the nature of adsorption processes as physical or chemical. The D-R isotherm equation is expressed as follows [50]:

(11) $$q_{e}=q_{D-R}e^{\beta \epsilon^{2}}$$

where *q*_*e*_ (mol/g) is the amount pollutions adsorbed on the adsorbent at equilibrium, q_D-R_ (mol/g) is the maximum adsorption capacity, β (mol^2^/J^2^) is a coefficient related to the mean free energy of adsorption, and ϵ (J/mol) is the Polanyi potential that can be written as:

(12) $$\epsilon =\mathrm{RT}\ln\left(1+\frac{1}{C_{e}}\right)$$

The constant *β* gives an idea about the mean free energy *E* (kJ/mol) of adsorption can be calculated using the relationship:

(13) $$E=\frac{1}{\sqrt{-2\beta }}$$

If *E* value is between 8 and 16 kJ/mol, the adsorption process follows by chemical ion-exchange and if *E* < 8 kJ/mol, the adsorption process is likely physical adsorption.

**Figure 11:**
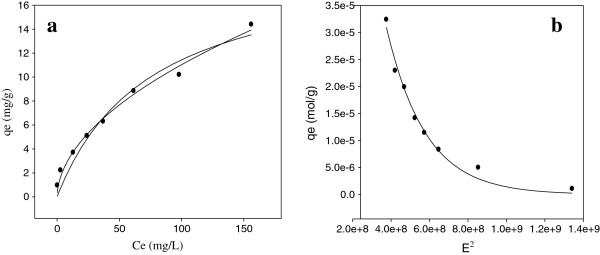
Langmuir, Freundlich (a) and D-R (b) isotherm models.

Adsorption isotherms for TC on pumice stone are given in Figure 11. The isotherm constants calculated from the Langmuir, Freundlich and D-R isotherm models and the correlation coefficients are given in Table 6.

**Table 6:** Langmuir, Freundlich and D-R adsorption isotherm parameters of TC

	**Langmuir**				**Freundlich**			**D-R**				
TC	Q_m_ (mg/g)	b (L/mg)	R_L_*	R^2^	k_F_ (L/g)	1/n	R^2^	q_D-R_ (mol/g)	q_D-R_ (mg/g)	E (kJ/mol)	β (mol^2^/J^2^)	R^2^
	20.02	0.0133	0.43	0.978	0.99	0.52	0.994	0.0002	90	10.08	4.92510^−9^	0.992

*R_L_ value calculated in 100 mg/L initial TC concentration is given.

The correlation coefficients of Langmuir (R^2^ 0.978), Freundlich (R^2^ 0.994) and D-R (R^2^ 0.992) models well fitted the adsorption data. The TC adsorption is compatible with both Langmuir and Freundlich isotherms. The applicability of both Langmuir and Freundlich isotherms on the adsorption of TC onto pumice stone shows that adsorption occurs under monolayer and heterogeneous surfaces. From Langmuir model, the maximum adsorption capacity (Q_m_) was determined to be 20.02 mg/g. Using the Freundlich model, k_F_ and 1/n were found to be 0.99 and 0.52, respectively. The 1/n heterogeneity value was between 0 and 1 indicating that the TC adsorption on the pumice stone was favorable at studied conditions.

The Q_m_ values of various adsorbents for TC antibiotics are presented in Table 7[1,3,19,25,51].

**Table 7:** **Maximum adsorption capacity (Q**_**m**_**) of various adsorbents for TC**

**Adsorbent**	**Q**_**m **_**(mg/g)**	**Reference**
Pumice stone	20.02	This study
Magnetite nanoparticles (Fe_3_O_4_ MnPs)	476	[1]
Graphene oxide	212	[3]
Montmorillonit	54	[19]
Activated sludge	72	[51]
Kaolinite	4.32	[25]

The calculated R_L_ values range between 0 and 1, indicating that the TC adsorption on pumice stone is favorable.

The mean free energy (E; kJ/mol) values calculated from the D-R isotherm model were also determined between 8 and 16 kJ/mol. It was indicating that the adsorption is mainly a chemical process occurs through cation exchange.

### Thermodynamic parameters

In order to explain the mechanism of TC adsorption onto pumice stone, the thermodynamic parameters of adsorption were calculated using free energy change (ΔG), enthalpy change (ΔH) and entropy change (ΔS) given by Equations 11 and 12 [1]:

(14) $$\mathrm{\Delta G}=-\mathrm{RT}\ln K_{C}$$

where *∆G* is the Gibbs free energy change, *R* is the universal gas constant (8.314 J/mol K), *T* is the temperature (K) and *K*_*C*_ (q_e_/C_e_) is the equlibrium constant.

The entalpy change (∆H) and entropy change (∆S) parameters were estimated from the following equation:

(15) $$\ln K_{C}=\frac{\Delta S}{R}-\frac{\Delta H}{\mathrm{RT}}$$

where the *∆H* and *∆S* in the biosorption process was determined from a slope and intercept of the plot of *ln K*_*C*_ versus *1/T*, respectively. The values of ∆H, ∆S and ΔG were calculated using equations 11 and 12 (Table 8).

**Table 8:** Thermodynamic parameters of TC adsorption on pumice stone

**Parameters**	**Temperature (K)**	**Adsorbent**
∆H (kJ/mol)		−4.53
∆S (kJ(molK)^−1^)		31.78
∆G (kJ/mol)	293	−13.79
	303	−14.23
	313	−14.47
	323	−14.77

The value of ΔH and ΔG were negative, indicating that the TC adsorption process is feasible, spontaneous and exothermic. The positive value of ∆S shows the increased in randomness at the solid/liquid interface during the adsorption process.

### The analysis of ions in aqueous solution after TC adsorption

The amounts of ions (Ca^2+^, Mg^2+^, Na^+^, K^+^ and Fe^3+^) in aqueous solution after TC adsorption at optimum conditions (pH 3.0, 50 mg/L TC concentration, 10 g/L pumice stone, 120 min, room temperature) were analyzed and the results were given in Table 9.

**Table 9:** The amounts of ions passing from pumice stone into TC solution

**Parameters**	**Concentration (mg/L)**
Ca^2+^	13.2
Mg^2+^	0
Na^**+**^	3.58
K^+^	10
Fe^3+^	4.59
Total ion amount	31.38

According to Table 9, total ion amount in aqueous solution after TC adsorption at optimum conditions is 31.78 mg/L. The amount of adsorbed TC by pumice stone at optimum conditions is 37.09 mg/g. In this case, a major part of TC adsorption on pumice stone realized with cation exchange between ions (Ca^2+^, Mg^2+^, Na^+^, K^+^ and Fe^3+^) in pumice stone with TC antibiotics.

## Conclusions

In this study, pumice stone was used as a new adsorbent for TC antibiotics removal from the aqueous solution. XRD, FT-IR and SEM data show that TC species adsorbed onto pumice stone. The adsorption mechanism of TC on pumice stone was cation exchange and surface complexation. The adsorption process is pH dependent and the optimum pH was found to be 3. The adsorption isotherm data for pumice stone could be fitted well by both Langmuir and Freundlich isotherm models. The maximum adsorption capacity (Q_m_) was found to be 20.02 mg/g. TC adsorption reached equilibrium within 90 min and adsorption kinetics fitted well to the pseudo-second order kinetic model. In the presence of Na^+^ cations, the TC adsorption decreased with increasing pH. The presence of Cu^2+^ was facilitated TC adsorption on pumice stone at low pH. Also, the TC adsorption onto pumice stone is found to be feasible, spontaneous and exothermic from thermodynamic studies.

The band changes of amide I or C = O amide groups in FT-IR spectra of TC adsorption on pumice stone was demonstrated that TC was adsorbed to the pumice stone with cation exchange and surface complexation.

The pumice stone was found to be an efficient adsorbent for the removal of TC antibiotics from aqueous solution. However, the effect of pretreatment for enhances the adsorption capacity of pumice stone can be examined in further studies.

## Competing interests

The authors declare that they have no competing interests.

## Authors’ contributions

The overall implementations of this study were the results of efforts by corresponding author. All authors have made contribution into the review and finalization of this manuscript. All authors read and approved the final manuscript.
